# Lower empathy for face mask wearers is not explained by observer’s reduced facial mimicry

**DOI:** 10.1371/journal.pone.0310168

**Published:** 2024-09-18

**Authors:** Sarah D. McCrackin, Jelena Ristic

**Affiliations:** Department of Psychology, McGill University, Montreal, Quebec, Canada; University of Bologna, ITALY

## Abstract

Facial occlusion alters social processes that rely on face visibility, including spontaneous mimicry of emotions. Given that facial mimicry of emotions is theorized to play an important role in how we empathize or share emotions with others, here we investigated if empathy was reduced for faces wearing masks because masks may reduce the ability to mimic facial expressions. In two preregistered experiments, participants rated their empathy for faces displaying happy or neutral emotions and wearing masks or no masks. We manipulated mimicry by either blocking mimicry with observers holding a pen in between their teeth (Experiment 1) or by producing a state of constant congruent mimicry by instructing observers to smile (Experiment 2). Results showed reduced empathy ratings for masked faces. Mimicry overall facilitated empathy, with reduced empathy ratings when mimicry was blocked and higher empathy ratings when it was instructed. However, this effect of mimicry did not vary with mask condition. Thus, while observers were impaired in sharing emotions with masked faces, this impairment did not seem to be explained by a reduction in facial mimicry. These results show that mimicry is an important process for sharing emotions, but that occluding faces with masks reduces emotion sharing via a different mechanism.

## Introduction

Face masks are one of the simplest and most effective ways for preventing contagion [1–3]. Despite clear medical benefits, visual face obstruction by face masks negatively affects multiple aspects of human nonverbal communication [4–7 for reviews] including person identification [8, 9], facial emotion recognition [e.g. 4, 9–12], emotion understanding [13, 14], and emotion sharing in the process of affective empathy [13]. Given these social impairments of visual facial occlusion, it is important to understand the mechanisms by which face occlusion affects our social communication. Understanding these mechanisms will both elevate our understanding of face perception mechanisms as well as inform policy development on how to improve communication during pandemics and in settings where visual occlusion with masks remains vital, such as healthcare [15–17].

Humans communicate emotions quickly via facial expressions [18–22], with different face parts transmitting information for different emotions [e.g. 21, 22]. As face masks cover about 60–70% of the face area we use to recognize emotions [e.g., 4], it is not surprising that humans are impaired at recognizing [e.g. 4, 9–12] and reasoning [13, 14] about the emotions of mask wearers. Face masks also exert a negative impact on the more complex socioemotional processes that depend on basic emotion perception, including our ability to share positive emotional states with mask wearers in the process of positive affective empathy [13].

Affective empathy refers to sharing another person’s emotional state, while understanding that the source of the emotion originates from that person [23–26]. In a recent study [13], participants viewed images of masked and unmasked face stimuli showing happy and sad emotional expressions and rated their level of empathy for the faces. Participants reported sharing less positive emotion (i.e., positive empathy) with masked faces, though sharing of negative emotions (i.e., negative empathy) was not affected by masks. Given that positive affective empathy is associated with higher prosociality [27], social competence [28], and increases the intrinsic reward value of relationships [27, 29], it is important to understand the mechanisms behind lower empathic responding to mask wearers. In this study we examined if reduced affective empathy for individuals wearing face masks may occur because face masks prevent observers’ spontaneous facial mimicry of the mask wearer’s facial expressions.

Facial mimicry refers to the imitation of the facial muscle positioning, or facial mirroring, that occurs in humans when we view faces displaying emotional expressions [30, 31]. For example, activation of the lip-raiser muscle, zygomaticus major, occurs in response to seeing happy faces. Facial mimicry occurs spontaneously, outside of conscious awareness [32] and cannot be willfully supressed through instruction [33]. Critically, facial mimicry is impaired in response to faces wearing masks [34, 35] and has been implicated in empathy. Individuals with higher trait affective empathy tend to display more facial mimicry [36–38], while instructing participants to mimic facial expressions increases state affective empathy for the expressor [39, 40].

There are at least two lines of evidence linking facial mimicry with empathy. First, increased mimicry has been associated with a better ability to recognize positive emotional states in others [e.g., 41, 42, but see 43 for null results], which is an important step towards sharing emotions. Reducing mimicry by blocking facial muscle movement [e.g., 42, 46] reduces recognition of happy expressions [41, 42] and genuine smiles [44], delays detection of the offset of smiles [45] and reduces the mu electroencephalography (EEG) desynchronization associated with recognizing happy expressions [46]. Given that affective empathy involves sharing another’s emotional state, recognition of their emotional expressions [47] is theorized to lead to the emotional state inferences [48] and perspective taking [49] that is often needed for an empathetic response. Accordingly, performance on emotion recognition tasks is linked to self-reported empathic concern [49], perspective taking [50], and trait empathy [51, 52], such that individuals who are found to be worse at identifying emotions from masked faces also report lower trait empathy [53] and empathic concern [54]. Together, these findings suggest that reduced mimicry of masked faces could impair empathy by preventing emotion recognition of happy expressions.

Second, facial mimicry and empathy have also been linked through embodiment [e.g., 55, 56] and facial feedback [57], which suggest that facial muscle activity generated by emotional mimicry results in neural feedback leading to associated feelings of mimicked emotional states [57, 58 for a review]. In other words, seeing a smile simulates a smile and the corresponding happy emotion via activation of emotional processing areas involved in feeling and sharing of positive emotions [59, 60]. In line with this, increased mimicry has been associated with greater amygdala [61, 62], striatum [61], and interior insula [62] activity, all of which are brain areas traditionally associated with emotional processing.

Given this evidence linking facial mimicry with empathy, we reasoned that facial occlusion by masks might reduce empathy by interfering with the spontaneous mimicry processes. We tested this notion in two preregistered experiments, in which we presented participants with images of faces showing positive or neutral facial emotions and wearing an opaque mask, a clear mask, or no mask. Critically, in separate Experiments, we either blocked facial mimicry by asking participants to hold a pen in their teeth (Experiment 1 [e.g. 41, 46]) or instructed mimicry by asking participants to smile throughout the task (Experiment 2 [e.g. 39, 40]). If empathy for masked faces is reduced because of observers’ reduced facial mimicry, keeping mimicry consistent by either preventing or enhancing it should reduce the difference between empathy ratings for masked and unmasked faces.

### Experiment 1

While affective empathy occurs for both negative and positive emotions (e.g., sharing sadness over a loss or happiness over a win [63]), we recently found that only positive affective empathy was reduced for masked face stimuli [13], which aligns with the findings showing that mimicry of happy expressions is impacted by face masks the most [34, 35]. Furthermore, our work also revealed that positive empathy was impaired for faces wearing opaque or clear face masks, despite clear masks restoring most of the visual access to the face and judgements about the faces’ affective state [13, see 64 for a review on clear masks]. Thus, restoring visual access to the face does not fully reinstate empathic responding to masked faces.

In Experiment 1, we investigated if reduced facial mimicry in observers may be a potential mechanism by which face masks lead to reduction in state empathic responses, to assess the links between emotional face perception and mimicry in emotional state sharing. To do so, we presented participants with images of face stimuli showing happy and neutral facial expressions. Faces wore opaque, clear, or no masks. In half of the trials, mimicry in observers was inhibited by instructing observers to hold a pen in their teeth, which prevents activation of the zygomaticus major (No Mimicry condition [e.g., 41, 46]). In the other half of trials, spontaneous mimicry in observers was allowed by providing no special instructions (Spontaneous Mimicry condition). If face masks impair empathy by reducing facial mimicry in observers, the impact of face masks on empathy ratings should be the greatest in the Spontaneous Mimicry condition where mimicry should occur more for unmasked than masked faces.

### Methods

#### Transparency and openness

Please see the Participants section for descriptions of our sample size, power calculations, and data exclusions. Data analysis was completed in SPSS 22. The study follows the Journal article reporting standards for quantitative research in psychology (JARS [65]). The experiment was pre-registered at https://osf.io/f4mvy. Anonymized and summarized data are available at https://osf.io/27ekt/, and analysis scripts are available upon request.

#### Participants

An a priori power analyses determined that data from approximately 60 participants were needed to obtain 90% power (at Alpha = .05) to detect a moderate size effect (*d* = .375) difference between empathy ratings for positive masked and unmasked faces as estimated from McCrackin et al. [13, 66]. Seventy participants (32 women, 36 men, 2 other; Mean age: 27.14, *SE* = 8.97) were recruited online from Prolific (https://app.prolific.com/studies) and were financially compensated. Preregistration procedures stipulated that any participants who did not complete more than 20% of the study trials or had more than 20% of trials removed for anticipatory responses (i.e. providing responses in less than 500ms) would be removed from analysis. No participants met these exclusion criteria, with an average number of 239.80/240 (SD = 0.47) valence and 239.76/240 (SD = 0.65) empathy ratings completed per participant. Participants were fluent in English, recruited from countries with significant exposure to Caucasian faces like those used as stimuli, had no psychological illness, no previous head trauma resulting in loss of consciousness, and reported normal or corrected-to-normal vision. The McGill University research ethics board approved this study.

#### Stimuli

The experiment was programmed online via Testable (https://www.testable.org/), which customizes the size of stimuli to fit each participant’s screen using a calibration procedure.

**Fig 1:**
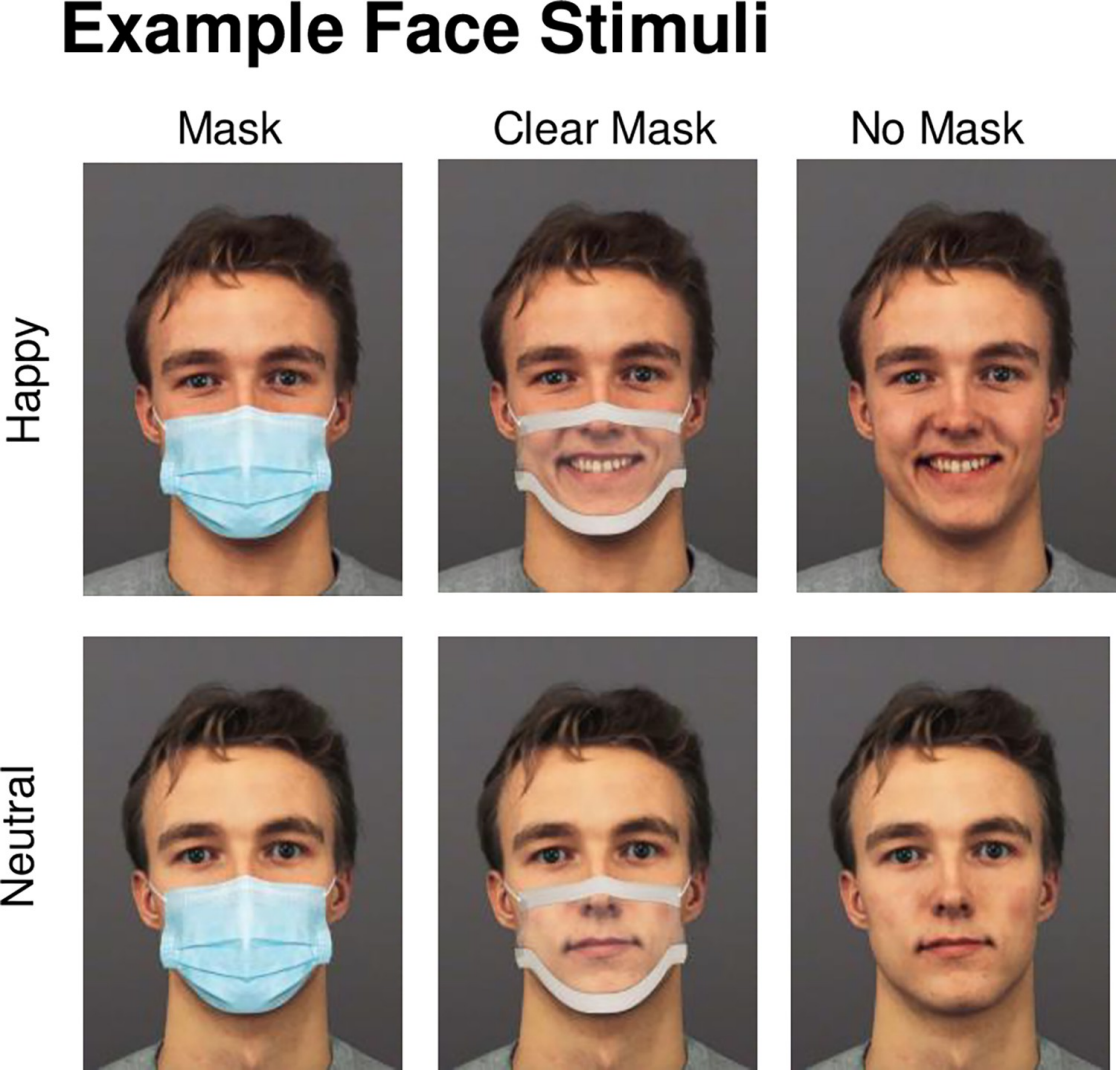
Example happy and neutral face stimuli with opaque, clear, or no mask. Face stimulus is modified and reprinted from the KDEF stimulus set (The Karolinska Directed Emotional Faces; [67]) under a CC BY license, with permission, original copyright [1998].

Fig 1 shows example stimuli. Images of 30 male and 30 female faces showing happy and neutral facial expressions were selected from the KDEF [67] and FACES [68] databases (**KDEF Identities:** KM08, KM11, KM31, KM35. **FACES Identities:** F05, F10, F13, F19, F22, F26, F28, F34, F40, F48, F54, F63, F69, F71, F85, F90, F98, F106, F115, F101, F125, F132, F134, F140, F162, F163, F171, F173, F177, F182, M08, M13, M16, M25, M37, M49, M57, M62, M66, M72, M81, M89, M99, M105, M109, M114, M119, M123, M127, M135, M144, M147, M153, M160, M170, M175. **Identities used in practice trials:** FACES identities F20 and M41). These stimuli have been independently validated for high emotional recognizability [68, 69]. Faces were fitted with opaque and transparent masks using Adobe Photoshop CS6. Masks were scaled to fit each face, spanning from the bridge of the nose to the lower chin, and between the edges of each cheek.

#### Design

The study was a repeated measures design with three factors, *Emotion* (2: Happy, Neutral), *Mask* (3: Opaque, Clear, No Mask), and *Mimicry* (2: No Mimicry, Spontaneous Mimicry,).

*Emotion* was manipulated between happy and neutral conditions by either showing a face with a happy emotional expression or a face with a neutral facial expression.

*Mask* manipulated whether the face was shown wearing an opaque mask, a clear mask, or no mask. The design and fit of each mask mirrored our previous work [13].

*Mimicry* was manipulated between No Mimicry and Spontaneous Mimicry. In the *No Mimicry* condition, participants were asked to hold a sanitized pen or pencil in between their teeth to block their ability to naturally mimic a smile [e.g., 41, 46]. This manipulation has reliably been shown to prevent activation of the muscles involved in smiling using electromyography [42] and to prevent mu desynchronization associated with recognizing happy expressions using electroencephalography [46]. Importantly, it does so without inducing an emotional state in the participant [45]. In the *Spontaneous Mimicry* condition, participants were asked to relax their face as usual, which allowed for spontaneous mimicry to occur.

Crossing the Emotion, Mask, and Mimicry factors yielded 12 experimental conditions. Each was repeated 20 times across 10 blocks. All factors were presented randomly, equally, and equiprobably. Emotion and Mask were intermixed while Mimicry was blocked, with half of the blocks randomly showing the No Mimicry condition and the other half showing the Spontaneous Mimicry condition. At the start of each No Mimicry block conditions, participants were shown an image of a face holding a pen between their teeth and were instructed, “*For the entire duration of this block*, *please position the object as shown below and hold it there while you complete the trials*.” At the start of each Spontaneous Mimicry conditions, participants were shown an image of a face with a neutral expression and no pen, paired with the instruction, “*For the entire duration of this block*, *please make sure the object is removed from your mouth and keep your face relaxed into its normal state*.”

The same face identities were presented in both the No Mimicry and the Spontaneous Mimicry conditions.

#### Procedure

**Fig 2:**
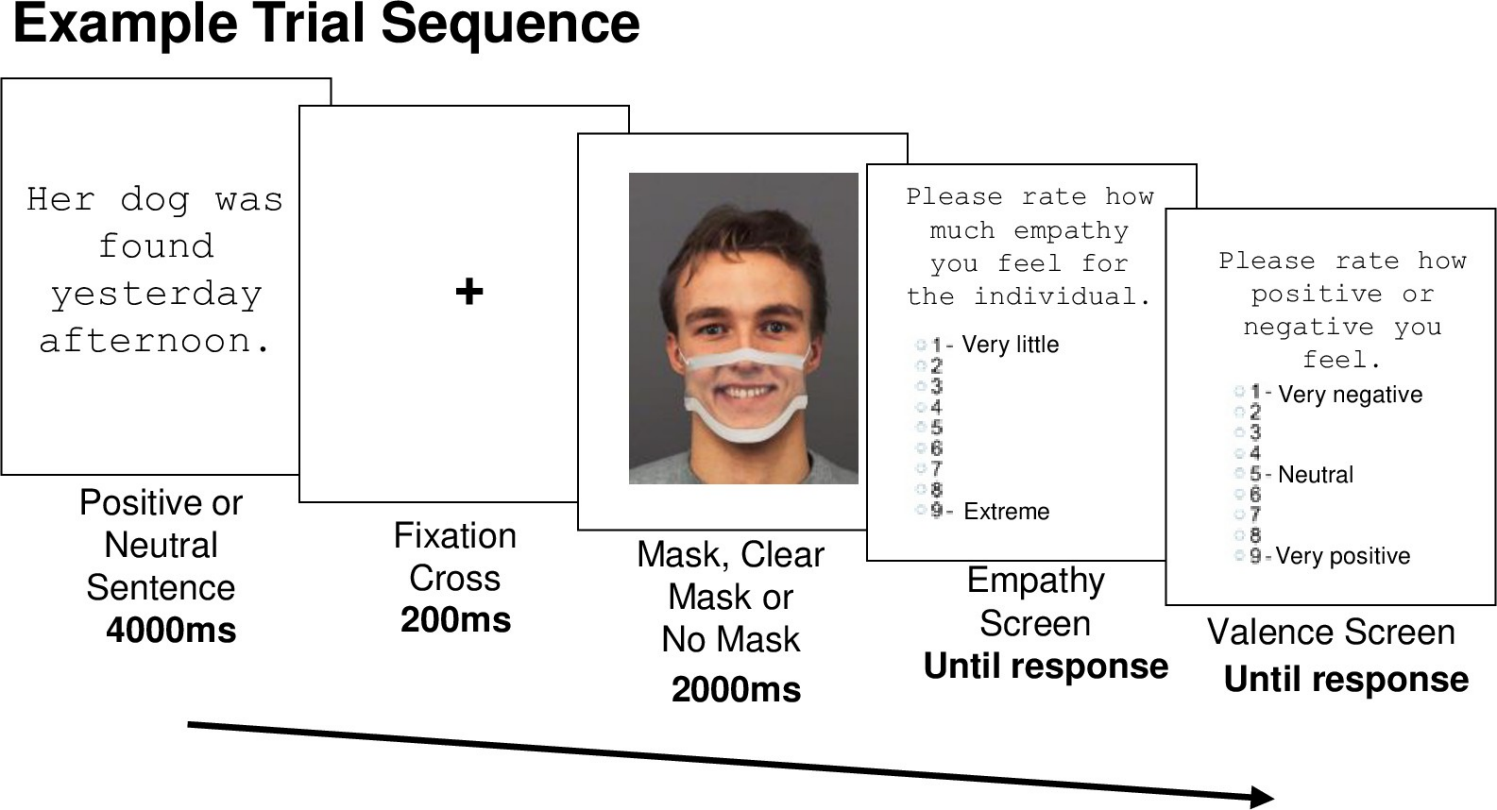
Example trial depicting a happy emotional expression with a clear mask. Face stimulus modified and reprinted from the KDEF stimulus set (The Karolinska Directed Emotional Faces; [69]) under a CC BY license, with permission, original copyright [1998].

Fig 2 illustrates an example trial sequence. Each trial began with a 4000ms presentation of a positive or neutral contextual sentence describing the face within a positive or neutral event (e.g., “Her dog was found/fed yesterday afternoon). The sentences have been validated previously [13, 14, 66, 70] and provide natural context for emotional processing. Twenty general sentence themes were selected, with the wording in each sentence matching the gender and emotion depicted on the face image (i.e., positive sentences paired with happy faces and neutral sentences paired with neutral faces). Pairing of contextual sentences and face identities was counterbalanced between participants. Next, a fixation cross in a duration of 200ms was shown, followed by a presentation of a face displaying happy or neutral facial expression, and wearing an opaque, clear, or no mask for 2000m.

In the present work, we were most interested in examining the impact of face occlusion on state empathy to assess the links between emotional face perception and mimicry in emotional state sharing. As this was a repeated measures design, participants served as their own empathy baseline, with scale responses examining how face visibility may affect those baseline ratings. For each face, participants first rated their Empathy “*Please rate how much empathy you feel for the individual*” using a Likert scale ranging from 1/Very little to 9/Extreme. These ratings provided a way to understand the amount of shared emotion with the face. Second, they rated Valence “*Please rate the valence of the emotion you are feeling*” using the same Likert scale ranging from 1/Very little to 9/Extreme. Valence ratings provided a corresponding measure of perceived emotional direction and qualia, verifying empathic response and emotional manipulation.

We have previously verified that these measures are related to trait empathy during the original task validation [66]. In this previous study, we collected trait affective empathy ratings using the Toronto Empathy Questionnaire (TEQ; [71]). As one would expect from a state measure of empathy [e.g. see 72], those with higher trait empathy reported stronger empathy ratings in response to the task, as measured with strong positive correlations. Following from this initial work, in the present study, empathy was defined for participants during the task instructions as “the sharing of someone’s emotional state while being aware that the emotion you are feeling is from the other person. Empathy can be both positive (e.g. feeling happy when you see a child laugh, and thus sharing in the child’s positive emotion) or negative (e.g. feeling sad when you see a child cry, and thus sharing in the child’s negative emotion).” Valence was also defined as “how positive or negative the emotion you are feeling is.”

Participants completed a total of 240 trials divided equally over 10 testing blocks. Three practice trials for each mimicry condition were run at the start.

## Results

Each participant’s empathy and valence ratings were averaged across the conditions of interest and examined using separate repeated-measures ANOVAs with Emotion (2: Happy, Neutral), Mask (3: Opaque, Clear, No Mask), and Mimicry (2: No Mimicry, Spontaneous Mimicry) included as factors. Any post-hoc tests reflect two-tailed paired t-tests corrected for multiple comparisons using the Bonferroni method. Greenhouse-Geisser corrected degrees of freedom are reported when Mauchly’s test of Sphericity was significant.

**Fig 3:**
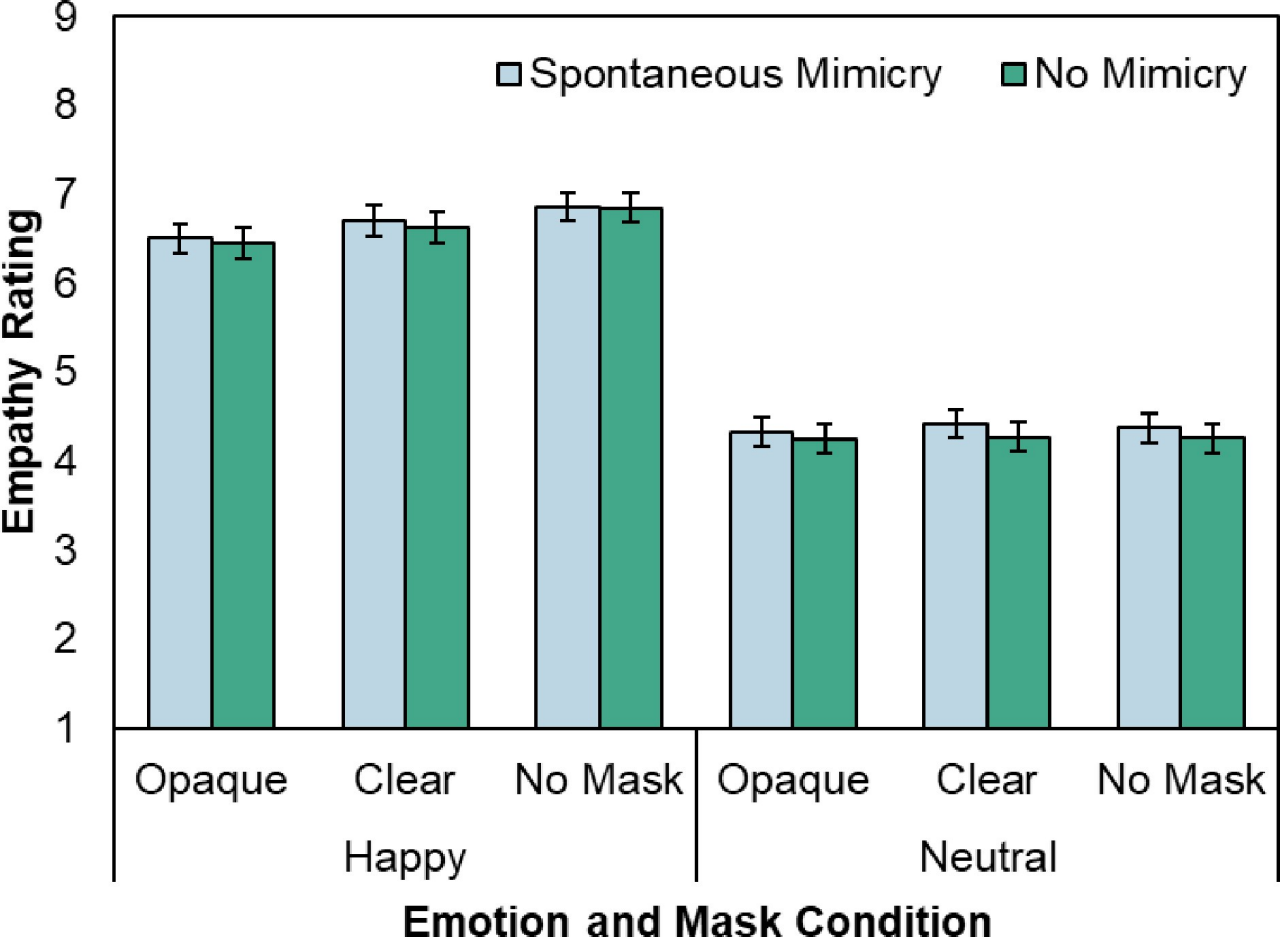
Mean empathy ratings as a function of emotion, mask, and mimicry. Error bars depict standard error of the mean.

Fig 3 plots the mean empathy ratings as a function of Emotion, Mask, and Mimicry. We hypothesized that if the impact of face masks on empathy could be explained by reduced facial mimicry, masks should impact empathy ratings more in the Spontaneous Mimicry condition, where more mimicry should occur for those without masks.

A significant main effect of Emotion (*F*(1, 69) = 202.63, *MSE* = 5.71, *p* < .001, *ηp^2^* = .75) confirmed that the emotional manipulation was successful. Participants reported more empathy (*p* < .001) in the happy condition compared to the neutral condition. Replicating our previous work [13], a reliable main effect of Mask (*F*(1.66, 114.48) = 5.41, *MSE* = .64, *p* = .005, *ηp^2^* = .073) further indicated that empathy ratings were lower when faces wore masks. A two-way interaction between Mask and Emotion (*F*(1.67, 115.31) = 12.28 *MSE* = .21, *p* < .001, *ηp^2^* = .15) showed that empathy impairment was larger for happy compared to neutral faces. Follow up ANOVAs on each emotion indicated that a main effect of Mask was found for happy faces (*F*(1.60, 110.54) = 9.33 *MSE* = .33, *p* < .001, *ηp^2^* = .12), but not for neutral ones (*F*(1.75, 120.64) = .59, *MSE* = .10, *p* = .56, *ηp^2^* = .008), where participants reported more empathy for unmasked faces compared to those wearing either opaque (*p* = .001) or clear masks (*p* = .012), which did not differ (*p* = .141).

There was also a main effect of Mimicry *F*(1,69) = 8.00, *MSE* = .17, *p* = .006, *ηp^2^* = .10, such that participants reported more empathy for faces in the Spontaneous Mimicry condition than in the No Mimicry condition. Critically, Mimicry did not interact with either Mask (Mask x Mimicry; *F*(2, 138) = 1.18, *MSE* = .054, *p* = .31, *ηp^2^* = .017) or Emotion (Emotion x Mask x Mimicry, *F*(2, 138) = .461, *MSE* = .051, *p* = .63, *ηp^2^* = .007; Emotion x Mimicry, *F*(1,69) = 2.04, *MSE* = .11, *p* = .16, *ηp^2^* = .029). The results for valence followed predicted patterns and replicated those for empathy. These data are presented in the S1 File.

Thus, while observers rated their empathy as higher when spontaneous mimicry was allowed to occur, and lower overall for faces wearing masks, either opaque or clear ones, Mimicry did not interact with mask condition to impact empathy ratings.

### Discussion

In Experiment 1 we found that participants reported more empathy and positive emotion during happy trials compared to neutral trials. Participants also reported more empathy and shared valence (see S1 File) in the condition which allowed spontaneous mimicry of the happy expressions to occur, compared to when mimicry was blocked with a pen, providing further support for the idea that spontaneous mimicry facilitates affective empathy [e.g., 41, 42, 57, 58]. Importantly, this finding also shows that our mimicry manipulation was effective.

Replicating our previous finding that masks impair positive empathy [13], reductions in empathy occurred both when faces wore opaque or clear masks. The reduction in empathy for clear masked faces suggests that the empathy reduction was not driven by lack of visual access to facial cues. However, since no interactions between Mask and Mimicry conditions were reliable, the findings from Experiment 1 further suggest that the reduction in empathy for mask wearers is likely also not driven by reduced facial mimicry in observers. In Experiment 2, we conceptually replicate this main result.

## Experiment 2

While Experiment 1 found that positive empathy was reduced for both clear and opaque face mask wearers, this impairment was not influenced by reduced facial mimicry. That is, while blocking mimicry by holding a pen between the teeth impaired empathy ratings overall, this manipulation did not interact with face masks.

It remains possible though that the mimicry manipulation was not highly effective. While this is unlikely, given that the use of the pen manipulation was reported in many previous studies [e.g., 41, 46] and given that reduced empathy is predicted from blocking mimicry [e.g., 39, 40], holding a pen between the teeth required participants to do something unusual. If so, a different mimicry manipulation may reveal an interaction with face masks.

In Experiment 2, we tested if a similar result would be obtained when using a different mimicry manipulation [39, 40]. In addition, we checked for task compliance by collecting half of the data in person. Thus, in Experiment 2, instead of blocking spontaneous mimicry, we actively instructed mimicry by asking participants to smile throughout. This provided conceptual replication of Experiment 1. If participants in Experiment 1 had lower empathy due to the pen manipulation being unusual or distracting, one would predict that participants in Experiment 2 would similarly have lower empathy for the smiling condition. In contrast, if the mimicry condition from Experiment 1 was effective, participants in Experiment 2 should report feeling more positive empathy when mimicking the expressions with a smile. Finally, and returning to the main questions, empathy should be generally enhanced during the Instructed Mimicry condition. If face masks impair empathy via blocked mimicry, we expected to find an interaction between mimicry and mask condition. In contrast, if face masks impair empathy via a different mechanism, we expected to replicate the lack of an interaction between face mask and mimicry condition from Experiment 1.

### Methods

#### Transparency and openness

Preregistration and data for Experiment 2 are available at https://osf.io/pz8cr and https://osf.io/27ekt/ respectively, and analysis scripts are available upon request.

#### Participants

Sixty-nine new naïve participants who were undergraduate students (60 women, 8 men, 1 other; mean age: 20.17, *SD* = 2.01) participated for course credit. Inclusion criteria were identical to Experiment 1. Participants completed on average 239.51/240 (*SD* = 1.20) empathy ratings and 239.43/240 (*SD* = 1.22) shared valence ratings. No participants were excluded from analyses. Thirty-four participants completed the experiment online and thirty-five participants completed the experiment in-person, under the supervision of a research assistant. The research assistant ensured that the smiling manipulation (see below) was adhered to by observing the participant throughout the test via a remote and discreet camera placed beside the observers computer screen. Participants were informed about this procedure.

#### Stimuli, design and procedure

Stimuli, design, and procedure were identical to Experiment 1, except that the *Mimicry* variable included *Instructed Mimicry* and *Spontaneous Mimicry*. In the Instructed Mimicry condition, participants were asked to smile while completing the trials to create a state of consistent and enhanced facial mimicry for the smiling faces. Participants were shown an image of a smiling face at the start of the block and were instructed, “*For the entire duration of this block*, *please smile and hold that smile while you complete the trials*.” The Spontaneous Mimicry blocks were identical to those in Experiment 1, with participants instructed to relax their face as usual, allowing for spontaneous mimicry to occur.

### Results

Data analysis mirrored Experiment 1, with average empathy and valence ratings examined using separate repeated measures ANOVAs with factors of Emotion (2: Happy, Neutral), Mask (3: Opaque, Clear, No Mask), and Mimicry (2: Spontaneous Mimicry, Instructed Mimicry) included as variables. Testing Location (2: Online, In-person) was entered as a between-subjects factor in each analysis and confirmed no reliable effects or interactions involving this variable (*Testing Location*, .11 < *ps* < .61), which confirmed that both online and in-person participants were compliant with the smiling manipulation. The analyses were re-run without this factor and reported below. Once again valence data replicated those for empathy and are reported in the S1 File.

**Fig 4:**
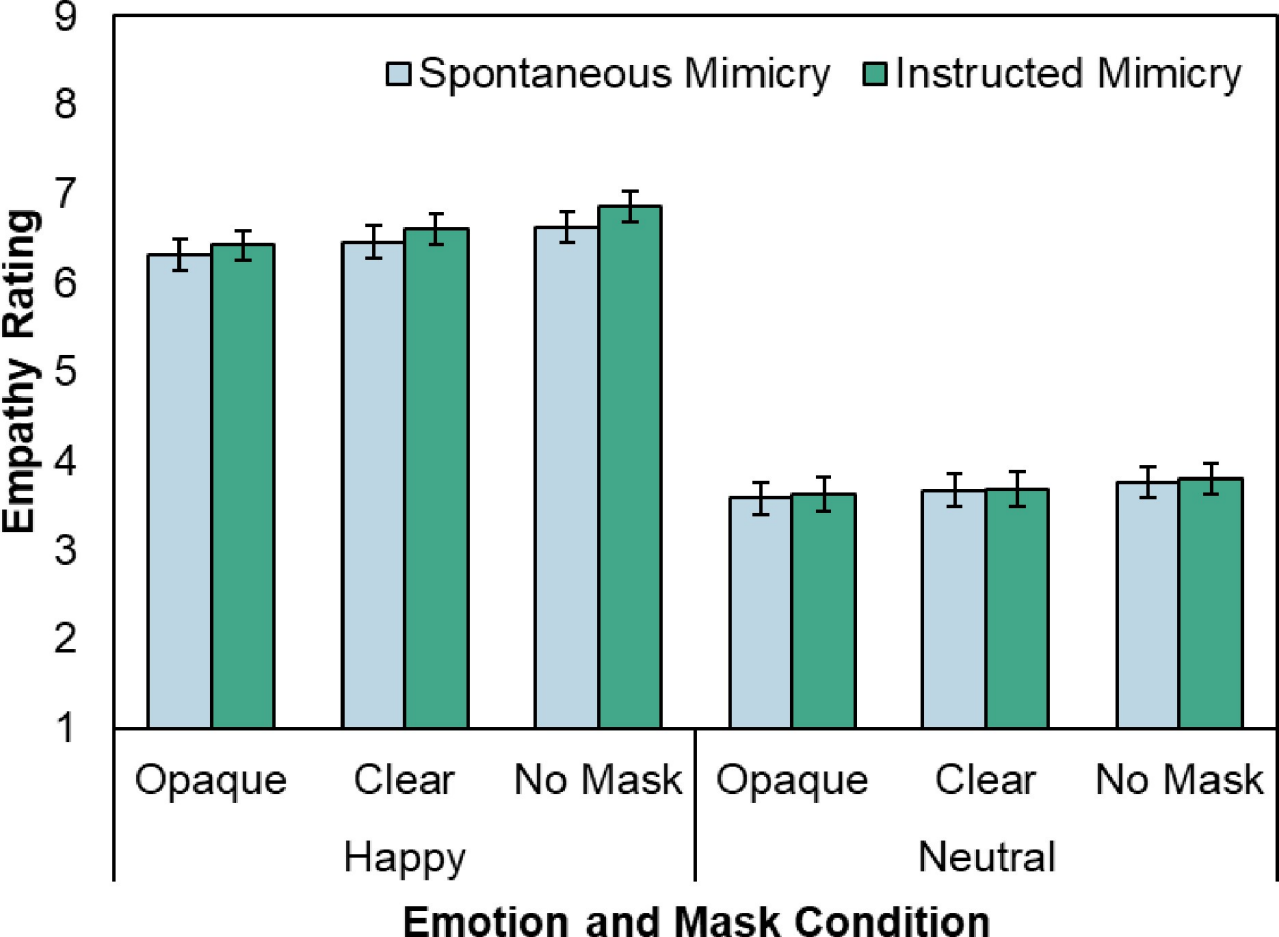
Mean empathy ratings as a function of mask, emotion, and mimicry. Error bars represent standard error of the mean.

Fig 4 illustrates the mean Empathy ratings as a function of Emotion, Mask, and Mimicry. We predicted that empathy would be generally enhanced during the Instructed Mimicry condition and reduced when faces wore clear or opaque masks. If face masks impair empathy via a different mechanism than facial mimicry, we expected to replicate the lack of an interaction between face mask and mimicry condition from Experiment 1. The data supported these predictions.

The analysis indicated a reliable main effect of Emotion (*F*(1,68) = 243.34, *MSE* = 6.98, *p* < .001, *ηp^2^* = .78) with higher empathy ratings for happy than for neutral trials. A main effect of Mask (*F*(1.50,102.29) = 15.95, *MSE* = .34, *p* < .001, *ηp^2^* = .19) as well as an interaction between Mask and Emotion (*F*(2,136) = 5.64, *MSE* = .11, *p* = .004, *ηp^2^* = .077) were reliable. While there were effects of Mask for both happy (*F*(1.21, 82.02) = 32.15, *MSE* = .1.08, *p* < .001, *ηp^2^* = .32) and neutral (*F*(1.79,121.86) = 6.34, *MSE* = .20, *p* = .003, *ηp^2^* = .085) conditions, in the happy trials, participants reported more empathy for face stimuli wearing no masks than for face stimuli wearing opaque (*p* < .001) or clear masks (*p* < .001), and more empathy for those wearing clear masks as opposed to those wearing opaque masks (*p* = .001). During neutral trials, participants reported feeling more empathy for those wearing no masks compared to those wearing opaque masks (*p* = .009), with a trend towards higher empathy for those with no masks compared to clear masks that did not reach significance (*p* = .085)

Participants also reported higher empathy in the Instructed Mimicry condition compared to the Spontaneous Mimicry condition (*F*(1, 68) = 4.13, *MSE* = .48, *p* = .046, *ηp^2^* = .057), though this was stronger during happy trials compared to neutral ones, as evidenced by an interaction between Mimicry and Emotion (*F*(1,68) = 5.72, *MSE* = .15, *p* = .020, *ηp^2^* = .078). Indeed, Instructed Mimicry was associated with higher empathy ratings in happy (*p* = .007) but not neutral trials (*p* = .56). Critically, and once again, the two-way interaction between Mimicry and Mask (*F*(2,136) = 1.72, *MSE* = .048, *p* = .18, *ηp^2^* = .025) as well as the three-way interaction between Mimicry, Mask, and Emotion (*F*(2, 136) = 1.69, *MSE* = .045, *p* = .19, *ηp^2^* = .024) were not reliable.

### Discussion

Experiment 2 found that participants reported more positive empathy during the happy trials, and that positive empathy was reduced when face stimuli wore clear or opaque masks. While instructing mimicry by asking participants to smile enhanced empathy ratings overall, no interactions with masks were statistically reliable, once again suggesting that wearing masks impairs empathy through a mechanism different from facial mimicry.

Importantly, Experiment 2 provided converging evidence for the notion that facial occlusion by masks impacts empathy independently of observers’ facial mimicry and rules out an alternative possibility that the mimicry manipulation in Experiment 1 was not effective. Indeed, while inhibiting mimicry in Experiment 1 resulted in impaired empathy, enhancing mimicry in Experiment 2 resulted in enhanced empathy. Given that both manipulations modulated empathy in opposite and predicted directions, it is reasonable to conclude that the observed mimicry effects are not due to one mimicry condition being unusual or distracting to participants.

## General discussion

A wealth of studies have recently reported that wearing face masks impacts human social interactions [5, 7] by lowering emotion recognition [e.g., 4, 10, 11] and the related processes of sharing emotional states with others [13]. Given that face masks remain important for preventing contagion [1–3], and that pandemic frequency is projected to rise [15–17], it is important to understand why empathy is reduced for faces wearing face masks. In the present study, we investigated this question by examining if empathy was impaired for mask wearers because of a reduction in observers’ facial mimicry. In two preregistered experiments, we presented participants with images of faces showing happy and neutral facial expressions and wearing opaque, clear, or no masks. We asked participants to rate their empathy and shared emotional valence for each face while their mimicry was either blocked (Experiment 1) or enhanced (Experiment 2).

First, the results replicated our previous finding showing that positive empathy was impaired for mask wearers [13]. Second, the results also verified that empathy reduction was not solely driven by visual face occlusion, as empathy was reduced similarly for faces wearing opaque masks and for faces wearing clear masks. Finally, and critically, blocked or enhanced facial mimicry did not modulate any of these effects. Next, we discuss two points related to these results.

First, our results show that facial mimicry is an important process for relating emotionally to others. In both experiments, participants reported more positive empathy during the conditions that maximized facial mimicry. In Experiment 1, more empathy was reported during the spontaneous mimicry condition when observers’ mimicry was unaltered compared to the condition in which their spontaneous mimicry was blocked using a pen manipulation [as in 41, 46]. Likewise, in Experiment 2, more empathy was reported when mimicry was instructed during the smiling condition [as in 39, 40] compared to the condition of unaltered spontaneous mimicry. These results add to and extend a body of research suggesting that facial mimicry and empathy are positively associated [36–40]. Facial mimicry has been theorized to facilitate affective empathy both by aiding in recognition of emotional states [e.g., 41, 42, 44, 46] and by contributing neural feedback associated with feeling the emotion that the other person is expressing [e.g. 55, 56, 59–61]. Our results align more with the neural feedback hypothesis since empathy was increased by mimicry regardless of whether emotional expressions were occluded by an opaque mask or visible behind a clear one. Thus, our findings highlight facial mimicry as an important social process for relating to those around us.

Second, and critically, the link between facial mimicry and empathy ratings was not affected by manipulation of facial occlusion. While previous work has reported that seeing individuals wearing face masks impairs observers’ mimicry of happy expressions [34, 35], the mimicry manipulations in the present study did not modulate the impact of face masks on empathy ratings. This suggests that while face masks may generally lower mimicry response in observers [34, 35], the mimicry reduction is likely not responsible for reduced empathy.

Furthermore, the mechanism by which reduced empathy occurs appears not to depend on the visibility of the face as both clear and opaque masks reduced empathy. It is possible that both opaque and clear face masks may prevent interpretation of facial cues via disruption of holistic face perception. This makes a prediction that the impact of masks on empathy may interact with manipulations of holistic face perception, such as face inversion [73] and could be neurally indexed by the N170 event-related potential as a holistic processing marker [74]. Wearing face masks may also create the perception of psychological distance between the wearer and the observer, which has been shown to impact empathic [75] responses. This predicts that manipulating feelings of psychological closeness [e.g., 76] may counteract the impact of face masks on empathy. These alternatives remain to be addressed with future research.

Future research is also needed to address some outstanding points. For example, our mimicry manipulations instructed participants to hold a pen in their mouth or maintain a smile. While we collected the data for half of our participants in Experiment 2 in person to ensure task instruction compliance (and found no difference in responses between the online and in-person participants), the bulk of our data collection was completed online where compliance with task instructions was unmonitored. Fully in person data collection would allow for better monitoring of task compliance and would further provide an opportunity to incorporate additional measures associated with mimicry responses, such as electromyography, for a more direct measure of observers’ mimicry, in turn providing a more detailed link to state empathy. Further, while our data were analyzed at a group level as a part of our preregistration plan, the link between facial mimicry and empathy has often been found to be affected by individual differences such as social competence [77] or cultural background [78]. Thus, while we controlled the amount of exposure to faces by restricting our sample to participants from countries with predominantly Caucasian face expertise, there may be subtle cultural differences in facial mimicry [78 for a review], which could potentially follow from cultural differences in emotion perception and communication [e.g. 79, 80]. Thus, future work could examine the links between mimicry and empathy as a function of cultural and other individual variables.

In sum, in the present study we found that while positive empathy was impaired for mask wearers and facilitated by facial mimicry, reduced facial mimicry in observers did not appear to be the reason for why those empathy ratings were reduced for mask wearers. Future research into the mechanism linking face occlusion by masks to empathy is critical to advance our theoretical understanding of emotional and empathic processing.

## Supporting information

S1 File Descriptions and analyses of valence ratings can be found in the Supplementary materials (SM). (DOCX)
