# Supplementary material for: Lower empathy for face mask wearers is not explained by observer’s reduced facial mimicry
Source: PLoS One. 2024 Sep 18;19(9):e0310168. doi: 10.1371/journal.pone.0310168 (PMC11410256; doi:10.1371/journal.pone.0310168)
Supplement: S1 File — (DOCX) [file pone.0310168.s001.docx]

**Supplementary Materials**

**For**

**Lower empathy for face mask wearers is not explained by observer’s reduced facial mimicry**

Sarah D. McCrackin and Jelena Ristic

*Department of Psychology, McGill University, Montreal, Quebec, Canada*

Address correspondence to:

Sarah McCrackin or Jelena Ristic

Department of Psychology

McGill University

2001 McGill College Avenue

Montreal, Quebec

H3A 1G1

Canada

Sarah McCrackin e-mail: [sarah.mccrackin@mail.mcgill.ca](file:///C:\Users\sarah\Desktop\STUDY%20INFO\GLASSES%20AND%20EMPATHY_ATOM\sarah.mccrackin@mail.mcgill.ca)

Jelena Ristic email: [jelena.ristic@mcgill.ca](file:///C:\Users\sarah\Desktop\STUDY%20INFO\GLASSES%20AND%20EMPATHY_ATOM\jelena.ristic@mcgill.ca)

**Experiment 1**

**Valence Ratings**

Figure S1 plots the mean ratings for Valence as a function of Emotion, Mask, and Mimicry. The results for Valence mirrored those for Empathy, supporting the main measure and manipulation of emotion.


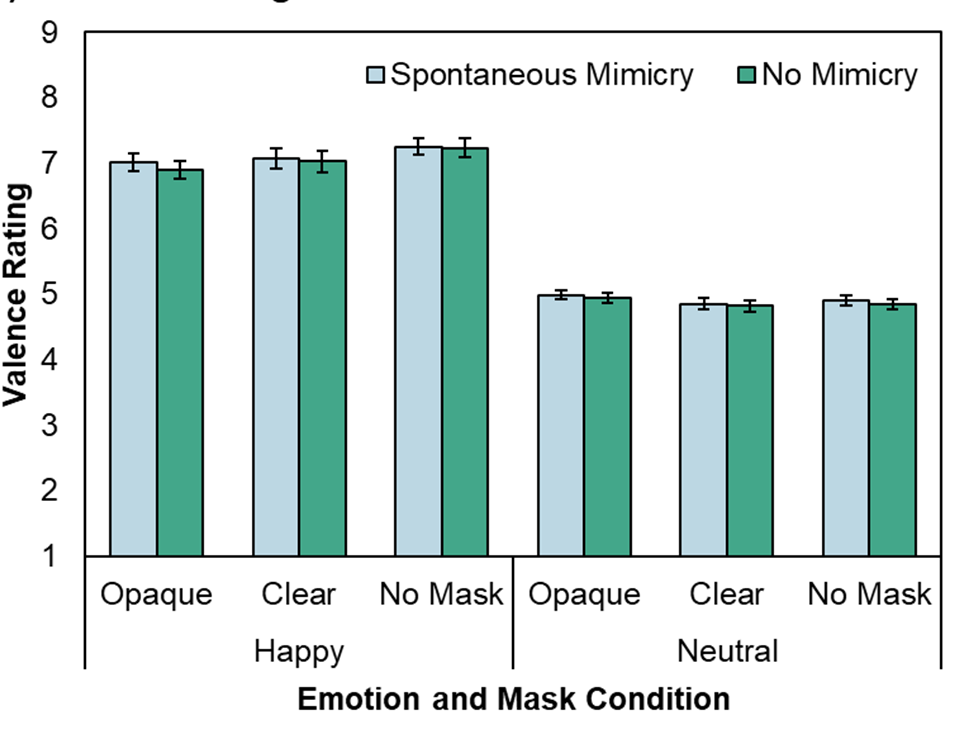


**Figure S1.** Mean valence ratings as a function of Mask, Emotion, and Mimicry. Error bars represent the standard error of the mean.

There was a reliable main effect of Emotion (*F*(1, 69) = 265.05, *MSE* = 3.79, *p* <.001, *ηp²* = .79) with more positive valence reported in the happy than neutral condition (*p*=.001). A main effect of Mask (*F*(1.31, 90.66) = 4.48, *MSE* = .38, *p* =.013, *ηp²* = .061) and a Mask x Emotion interaction, *F*(2, 138) = 18.04, *MSE* = .15, *p* <.001, *ηp²* = .21, supported reliable effects of masks on both happy (*F*(1.50,103.22) = 9.49, *MSE* = .22, *p* <.001, *ηp²* = .12) and neutral (*F*(2, 138) = 9.27, *MSE* = .034, *p* <.001, *ηp²* = .12) trials, replicating our previous findings (McCrackin et al. 2022b). In the happy condition, participants reported more positive valence in response to face stimuli wearing no masks compared to face stimuli wearing opaque masks (*p*<.001). While participants also reported more positive valence for faces wearing no masks compared to clear masks, the contrast did not survive the Bonferroni correction (*p*=.065, uncorrected *p=.*022). In the neutral condition, participants reported more positive valence for faces wearing opaque masks compared to those wearing clear (*p*<.001) or no masks (*p*=.009), which did not differ (*p*=.79).

There was also a main effect of Mimicry (*F*(1,69) = 4.69, *MSE* = .12, *p* =.034, *ηp²* = .06). Participants reported more positive valence towards faces in the Spontaneous Mimicry than in the No Mimicry condition. Once again, and critically, no interactions between Mimicry, Mask, and Emotion were found (Mask x Mimicry *F*(2, 138) = 1.13, *MSE* = .037, *p* =.33, *ηp²* = .016, Emotion x Mask x Mimicry *F*(2, 138) = 1.33, *MSE* = .038, *p* =.27, *ηp²* = .019; Emotion x Mimicry *F*(1,69) = .17, *MSE* = .10, *p* =.69, *ηp²* = .002).

Thus, while observers rated their shared valence as less positive when individuals wore masks, and more positive during the Spontaneous Mimicry condition, the mimicry condition did not modulate the mask effect.

**Experiment 2**

**Valence Ratings**

Valence results mirrored the empathy results in Experiment 2 and are plotted in Figure S2.


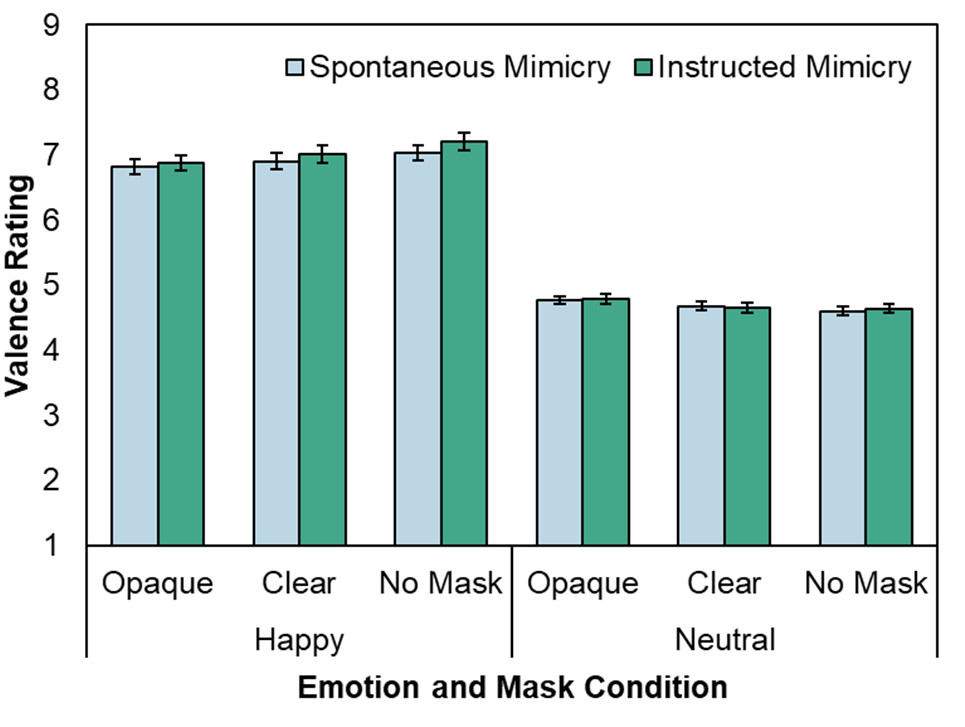


**Figure S2.** Mean valence ratings as a function of Mask, Emotion, and Mimicry. Error bars represent the standard error of the mean.

There were main effects of Emotion (*F*(1, 68) = 338.18, *MSE* = 3.21, *p* <.001, *ηp²* = .83) with more positive valence for happy trials than neutral trials. A main effect of Mask *F*(2,136) = 4.40, *MSE* = .063, *p* =.014, *ηp²* = .061, and a Mask by Emotion interaction (*F*(2,136) = 35.84, *MSE* = .085, *p* <.001, *ηp²* = .35) replicated the finding that masks reduce shared positive valence. During positive trials, participants reported more positive valence for unmasked faces compared to those with opaque (*p*<.001) or clear masks (*p*=.002), and more positive valence in response to clear compared to opaque masked faces (*p*=.003). During neutral trials, participants reported more positive valence for those wearing opaque masks compared to those wearing clear (*p*<.001) or no masks (*p<*.001).

Finally, while there was no main effect of Mimicry (*F*(1, 68) = 2.56, *MSE* = .32, *p* =.12, *ηp²* = .036), an interaction between Mimicry and Emotion (*F*(1, 68) = 4.71, *MSE* = .12, *p* =.033, *ηp²* = .065) indicated an increased positive valence in the Instructed Mimicry condition compared to the Spontaneous Mimicry condition for happy (*p*=.036) but not neutral (*p*=.76) trials. Again, there were no interactions between Mimicry and Mask (*F*(2, 136) = 2.49, *MSE* = .037, *p* =.087, *ηp²* = .035), or between Mimicry, Mask, and Emotion (*F*(2, 136) = 1.25, *MSE* = .44, *p* =.29, *ηp²* = .018).

To summarize, observers rated their shared valence as less positive when individuals wore clear or opaque masks compared to no masks, and more positive during the Instructed Mimicry condition. Once again, mimicry condition did not modulate the effect of masks on valence ratings.
